# Effects of noise exposure on young adults with normal audiograms I: Electrophysiology

**DOI:** 10.1016/j.heares.2016.10.028

**Published:** 2017-02

**Authors:** Garreth Prendergast, Hannah Guest, Kevin J. Munro, Karolina Kluk, Agnès Léger, Deborah A. Hall, Michael G. Heinz, Christopher J. Plack

**Affiliations:** a Manchester Centre for Audiology and Deafness, University of Manchester, Manchester Academic Health Science Centre, M13 9PL, UK; b Audiology Department, Central Manchester University Hospitals NHS Foundation Trust, Manchester Academic Health Science Centre, Manchester, M13 9WL, UK; c National Institute for Health Research (NIHR) Nottingham Hearing Biomedical Research Unit, Nottingham, NG1 5DU, UK; d Otology and Hearing Group, Division of Clinical Neuroscience, School of Medicine, University of Nottingham, Nottingham, NG7 2UH, UK; e Department of Speech, Language, & Hearing Sciences and Biomedical Engineering, Purdue University, West Lafayette, IN 47907, USA; f Department of Psychology, Lancaster University, Lancaster, LA1 4YF, UK

**Keywords:** Cochlear synaptopathy, Hidden hearing loss, Noise-induced hearing loss, Auditory brainstem response, Frequency-following response, ABR, auditory brainstem response, FFR, frequency following response, NIHL, Noise-induced hearing loss, OHC, outer hair cell, IHC, inner hair cell, AN, auditory nerve, SR, spontaneous rate, TEOAE, transient-evoked otoacoustic emission

## Abstract

Noise-induced cochlear synaptopathy has been demonstrated in numerous rodent studies. In these animal models, the disorder is characterized by a reduction in amplitude of wave I of the auditory brainstem response (ABR) to high-level stimuli, whereas the response at threshold is unaffected. The aim of the present study was to determine if this disorder is prevalent in young adult humans with normal audiometric hearing. One hundred and twenty six participants (75 females) aged 18–36 were tested. Participants had a wide range of lifetime noise exposures as estimated by a structured interview. Audiometric thresholds did not differ across noise exposures up to 8 kHz, although 16-kHz audiometric thresholds were elevated with increasing noise exposure for females but not for males. ABRs were measured in response to high-pass (1.5 kHz) filtered clicks of 80 and 100 dB peSPL. Frequency-following responses (FFRs) were measured to 80 dB SPL pure tones from 240 to 285 Hz, and to 80 dB SPL 4 kHz pure tones amplitude modulated at frequencies from 240 to 285 Hz (transposed tones). The bandwidth of the ABR stimuli and the carrier frequency of the transposed tones were chosen to target the 3–6 kHz characteristic frequency region which is usually associated with noise damage in humans. The results indicate no relation between noise exposure and the amplitude of the ABR. In particular, wave I of the ABR did not decrease with increasing noise exposure as predicted. ABR wave V latency increased with increasing noise exposure for the 80 dB peSPL click. High carrier-frequency (envelope) FFR signal-to-noise ratios decreased as a function of noise exposure in males but not females. However, these correlations were not significant after the effects of age were controlled. The results suggest either that noise-induced cochlear synaptopathy is not a significant problem in young, audiometrically normal adults, or that the ABR and FFR are relatively insensitive to this disorder in young humans, although it is possible that the effects become more pronounced with age.

## Highlights

• Large study on the effects of lifetime noise exposure in normal-hearing young adults.
• No clear evidence for noise-induced cochlear synaptopathy in ABR or FFR measures.
• Noise exposure associated with elevated 16-kHz audiometric thresholds in females.

## 1 Introduction

The primary account of noise-induced hearing loss (NIHL) is that cochlear hair cells are damaged (Liberman and Dodds, 1984), causing a loss of sensitivity to quiet sounds. This loss of sensitivity can be detected by pure tone audiometry, and thus NIHL can be identified by comparing thresholds to age-matched normal audiograms. Recently, experiments conducted in rodent models have demonstrated another mechanism of NIHL, cochlear synaptopathy, which is characterized by a loss of the synapses between inner hair cells (IHCs) and auditory nerve (AN) fibers. Using a mouse model, Kujawa and Liberman (2009) demonstrated that after 2 h of exposure to 100 dB SPL noise (8–16 kHz), up to 50% of the synapses between IHCs and AN fibers had been permanently destroyed in the affected frequency region. This permanent loss of AN synapses was seen despite a recovery in absolute sensitivity. Their results suggest that cochlear synaptopathy can be identified from a reduction in the amplitude of wave I of the auditory brainstem response (ABR), which reflects AN function. The reduction was only observed in response to moderate-to-high-intensity stimuli, not for stimuli presented near threshold.

Cochlear synaptopathy has been demonstrated in a number of other rodent models (e.g. guinea pig, Lin et al., 2011; chinchilla, Hickox et al., 2015) and has been shown to occur after exposure to more moderate sound levels over a longer duration (84 dB SPL for a week, Maison et al., 2013). Furthermore, noise-induced synaptic loss has been shown to preferentially affect the synapses with low spontaneous-rate (SR) AN fibers (Furman et al., 2013). Low-SR fibers have high thresholds and high saturation levels, and so are used to encode high-intensity sounds. Hence, noise-induced cochlear synaptopathy could result in coding of supra-threshold sounds being affected despite sensitivity near threshold remaining unaltered. The low-SR account of how synaptopathy manifests in rodents appears straightforward and well understood, however there are still unresolved issues. For example Song et al. (2016) demonstrated that, after noise exposure, synapses can remain present but are no longer functionally normal.

Currently, the most direct evidence for noise-induced synaptopathy occurring in humans is from a study demonstrating that the amplitude of wave I of the ABR in response to high-intensity clicks was negatively correlated with noise exposure across 30 participants, despite little effect of exposure on absolute threshold up to 8 kHz (Stamper and Johnson, 2015a). The measure of noise exposure quantified the amount of high-intensity sound encountered over the previous 12 months, rather than lifetime exposure. Hence, some listeners may have been classified as low noise exposed, when in fact earlier noise exposure may have already caused synaptopathy. Furthermore there was a confound due to the distribution of sexes across the cohort: Male participants formed the majority of the highly noise exposed listeners, and males tend to show weaker ABRs than females due to factors such as head size. This was subsequently addressed with separate analyses for males and females (Stamper and Johnson, 2015b), though this information was presented only for the highest sound level tested (90 dB nHL), and the authors did not confirm that there was no relation between hearing threshold and noise exposure separately for the two sexes. This re-analysis found a significant decrease in ABR wave I amplitude as a function of noise exposure for females, but not for males.

A more recent study by Liberman et al. (2016) found no significant decrease in wave I amplitude (“action potential”) measured from the ear canal in a group of listeners with normal audiometric thresholds identified as high-risk for noise-induced synaptopathy compared to a low-risk group. The authors do report a significant increase in the ratio of the summating potential (reflecting hair cell activity) to the action potential in the high-risk group, consistent with synaptopathy. However this increase in ratio was driven mainly by an increase in the summating potential in the high-risk group rather than by a decrease in the action potential in the high-risk group. Based on the studies of synaptopathy in rodents it was predicted that the summating potential would remain equivalent between the two groups. Hence, interpretation of this finding is not straightforward.

Attenuated wave I amplitudes have been observed in audiometrically normal human listeners with tinnitus compared to controls when hearing thresholds were matched between the groups (Schaette and McAlpine, 2011). Gu et al. (2012) also showed attenuated wave I amplitudes in tinnitus listeners compared to non-tinnitus controls, however the groups also differed in audiometric threshold above 8 kHz. Cochlear synaptopathy has been suggested as a possible cause of tinnitus in listeners with normal audiograms, with the percept arising from the auditory system trying to compensate for reduced AN input by increasing central neural gain. However, to the authors' knowledge, no published study has measured noise exposure and electrophysiological responses in the same human listeners with tinnitus and so it remains unclear the extent to which tinnitus is a symptomatic manifestation of noise-induced synaptopathy.

Wave I of the ABR is the most direct non-invasive measure of AN fidelity in humans, and in the rodent model has been shown to be a correlate of underlying cochlear synaptopathy, at least at the group level. However, one of the obstacles for the use of the ABR to identify synaptopathy in humans is that wave I amplitude is highly variable across individuals. Another objective measure that has been proposed as an indicator of synaptopathy is the frequency-following response (FFR). The FFR is a sustained evoked potential, reflecting neural phase locking to the fine structure or envelope of sounds. For frequencies from about 80 to 1000 Hz, the latency of the FFR is consistent with a generator in the rostral brainstem (Krishnan, 2006). Shaheen et al. (2015) demonstrated that the FFR may be a more robust indicator than the ABR of noise-induced synaptopathy in mice. Furthermore the FFR has been shown to relate reliably to behavioral performance on temporal discrimination tasks, which provides further evidence of the suitability of the FFR to detect noise-induced changes in neural processing (Bharadwaj et al., 2015).

The evidence for noise-induced synaptopathy in a range of rodent models is compelling. However, to date, evidence for noise-induced synaptopathy in humans is limited and it is unclear whether the same mechanism is involved in both males and females. Many of the rodent studies use male animals and sex has not been studied as a factor. Therefore, it remains unknown the extent to which the two sexes are equally susceptible to noise induced synaptopathy. If the pathology does occur in humans, we hypothesize that noise exposure will reduce the number of functioning low-SR AN fibers in the affected frequency region, leading to a reduction in the ABR response at high levels (specifically for wave I), and a reduction in the FFR at high carrier frequencies. The choice of stimuli for this study was informed by previous work in both rodents and humans and the approach assumes that synaptopathy will preferentially affect low-SR fibers and that the effects will be most readily observed in the 3 to 6 kHz characteristic frequency region where noise damage in humans is usually manifest (Toynbee, 1860, McBride and Williams, 2001).

In the present study, these measurements were compared to lifetime noise exposure. For both the ABR and the FFR two stimuli were used, the response to one of which was predicted to be more affected by noise-induced synaptopathy than the other. The ABR assumed to be most affected was that to a high-intensity click. This was compared to the ABR to a lower-intensity click that should have produced less activation of low-SR fibers. The bandwidth of the ABR stimuli was chosen to target the 3 to 6 kHz characteristic frequency region where NIHL is usually observed in humans (Toynbee, 1860, McBride and Williams, 2001). The FFR assumed to be most affected was that to the envelope of a 4-kHz carrier frequency. This was compared to an FFR for a low frequency pure tone (see Barker et al., 2014 for a preliminary use of this approach). The purpose of using such differential measures is to isolate the effects of synaptopathy from individual differences due to unrelated factors such as head size, and background physiological noise (see Plack et al., 2014, Plack et al., 2016 for further discussion).

## 2 Methods

### 2.1 Participants

One hundred and twenty six participants (75 females), with a wide range of noise exposures, were tested. All participants had audiometric thresholds within the normal range at octave frequencies from 500 to 8 kHz. Males had a mean age of 23.3 years (range, 18–36) and females had a mean age of 22.9 years (range, 18–36). The procedures were approved by the University of Manchester Research Ethics Committee and all participants gave informed consent (project number 14163).

### 2.2 Noise exposure

Lifetime noise exposure was estimated using a questionnaire developed to assess the effectiveness of the UK noise at work regulations (Lutman et al., 2008). The technique uses pre-determined categories such as “*clubs with amplified music*”, “*live amplified music*”, “*music through speakers*” and also considers miscellaneous activities which constitute a significant source of noise exposure for a given individual (for example playing in bands, attending live sporting events). The questionnaire considers both social and occupational noise exposures. For each activity, in each category, the duration and frequency of exposure is estimated from discussion with the participant and entered into the following formula:

$$U=10^{\left(L-A-90\right)/10}x\;Y\;x\;W\;x\;D\;x\;\mathrm{H/}\mathrm{2080}\text{,}$$

where *U* is cumulative noise exposure, *L* is estimated noise exposure level in dBA, *A* is hearing protection in dB, *Y* is years of exposure, *W* is weeks of exposure per year, *D* is days of exposure per week, *H* is hours of exposure per day, and 2080 corresponds to the number of hours in a working year.

The specific implementation of the noise exposure questionnaire used for our study differed from the procedure detailed in the original research report in a number of ways. In Lutman et al. (2008) activities with exposures estimated to be greater than 81 dBA were considered and the overall noise exposure unit was taken as the greatest noise exposure at the individual category level. We considered activities with exposures estimated to be greater than 85 dBA (this value represents the first action level for hearing protection as stipulated by the UK noise at work regulations) and noise exposure calculations were summed over all categories (social and occupational, current and historical). For our cohort the most common activities were attending nightclubs, attending live music events and playing in bands, all of which were assigned an estimated noise level of 105 dBA. There is large variability in the reported sound levels experienced in a nightclub, at a rock concert and by practicing musicians (see Smeatham, 2002 for a thorough overview). Despite the variability, it is clear that in such venues sound levels can reach an equivalent exposure in excess of 105 dBA (Stone et al., 2008) and so this level was selected as a reasonable estimate of sound levels encountered by our cohort when playing in bands, and attending amplified music concerts and nightclubs. Another common activity was listening to music via headphones. Estimating the sound level delivered to the ear by listening to portable devices is difficult due to the variability introduced by the device, the specific headphones used and the extent to which the headphones have decreased in efficiency over time. Commonly reported maximum output values are 97–107 dBA, with an average around 100 dBA (Portnuff et al., 2013). For the current study, participants were asked to imagine walking down the a busy high street and to describe whether they preferred to a) hear nothing except their own music, b) be generally aware of what is going on around them, such as traffic and sirens, but to be able to clearly hear their music over people talking around them, or c) hear everything that is present in the environment as they do not like having their sense of awareness compromised by their music. Listeners found it easy to relate to these conditions and listening values of 93 dBA and 87 dBA were reasonably assigned to preferences *a* and *b*, with the listening habits of category *c* not documented further. Background noise on a busy high street was assumed to be 80 dBA when determining these categories. It is conceivable that these estimated levels do not encompass the loudest listening levels used by some participants (those with the most extreme listening preferences in conjunction with music players and headphones capable of high intensity output). However this would not be expected to cause a major underestimation of their overall noise exposure unless such participants were regular listeners of loud music but *not* regular attendees of concerts and nightclubs. Listening preferences such as these were rare in the sample.

Estimated noise levels for different activities were fixed across participants to try to reduce the degree of error from subjective recall of noise levels. The majority of participants had never worked in a noisy environment and the main, and often only, category contributing to their noise score was “social noise exposure.” A subset of participants worked in the music industry in some capacity, either as professional musicians or as sound technicians. These participants reported significant noise exposure at work and many of these individuals form the upper tail of the noise exposure distribution.

One noise exposure unit is equivalent to exposure for 1 year to a working daily level of 90 dBA. For our purposes, we used the raw noise immission units and these were log transformed to produce a normal distribution. Each such logarithmic unit is equivalent to a factor of ten in terms of lifetime exposure energy.

### 2.3 Pure tone audiometry

Pure tone audiometry was performed in each ear separately at octave frequencies between 250 Hz and 8 kHz in accordance with the British Society of Audiology (2011) recommended procedure. Thresholds were measured using VIASYS GSI-Arrow audiometers coupled to TDH39P supra-aural headphones. The criterion for inclusion in the study was audiometric thresholds <25 dB HL in both ears at all frequencies.

High-frequency audiometry was also performed at 16 kHz using a Creative E-MU 0202 or 0204 USB soundcard. Sounds were played over Sennheiser HDA 200 circum-aural headphones designed for high-frequency audiometry. The sound stimulus was a quarter-octave band of noise centered at 16 kHz and converted from digital to analog at a sample rate of 48 kHz using a 24-bit depth. Stimuli were 220 ms in duration (including 10-ms raised-cosine ramps) ramps and there was an inter-stimulus interval of 500 ms. A three-alternative forced-choice procedure was used, with a two-down, one-up staircase adaptively setting the stimulus level. Stimulus level was varied arithmetically using a step size of 4 dB for the first four reversals and 2 dB for the following 10 reversals. Thresholds were calculated by averaging the final 10 reversals from a single run. 16-kHz hearing sensitivity was assessed to determine if high-frequency hearing could act as an early indicator of damage to the auditory system, before any effects are seen in the standard audiometric range.

### 2.4 Otoacoustic emissions

Transient evoked otoacoustic emissions (TEOAEs) were recorded using an ERO SCAN (Maico) screening system in order to evaluate listeners' outer hair cell (OHC) function. Six frequencies were tested in the range 1.5–4 kHz in 500 Hz steps using narrow band clicks presented at 83 dB peak-equivalent SPL (peSPL, defined as the level of sinusoid with the same peak-to-trough amplitude). Signal-to-noise ratios (SNRs) were obtained at the six test frequencies in both ears and for the purpose of analysis the SNR was averaged between the ears for the three test frequencies between 3 and 4 kHz. Due to technical difficulties, TEOAEs were only acquired on 79 of the 126 individuals included in the main EEG and audiological analyses.

### 2.5 Electrophysiology

#### 2.5.1 Recordings

All EEG recordings were made in a single 2-h session and used an ActiveTwo system (Biosemi, Amsterdam). Active electrodes were placed at the high forehead (Fz), the seventh cervical vertebra (C7) and the left and right mastoids (M1, M2). The potentials at all four individual electrodes were recorded at a sampling frequency of 16384 Hz, with differential montages constructed offline. No online filtering was applied (aside from the anti-aliasing filter implemented in hardware) and no online rejection criteria were set. Electrode offsets were maintained within ±30 mV throughout each recording, except for the ABR recordings from three participants in which one of the electrodes became detached during the recording (data from the affected channels were discarded). Recordings were made with the participant reclined on a chair and free to close their eyes and relax or fall asleep.

All stimuli were generated using MATLAB and presented through a Creative E-MU 0204 USB soundcard using a sampling frequency of 48 kHz with 24-bit resolution. Stimuli were presented using mu-metal shielded ER3A inserts (Etymotic, IL, USA). The sound card was used to send triggers to the Biosemi acquisition software to ensure that data collection and stimulus presentation were synchronized.

#### 2.5.2 ABR stimuli

Stimuli were 100-μs diotic clicks high-pass filtered at 1.5 kHz (using a fourth order Butterworth filter) and presented in alternating polarity. Because of the low-pass characteristic of the ER3A inserts, the stimulus delivered to the ear had a restricted bandwidth with a spectral plateau from about 1.5 to 4 kHz. Click levels were 80 and 100 dB peSPL (measured at the output of the inserts using an IEC711 2-cc coupler). Diotic clicks were used in an attempt to measure the strongest ABR possible from each listener. Presentation rate was 11 clicks/s and stimuli were interleaved such that 34 s of one click intensity were followed by 34 s of the other click intensity in order to ensure that any variability across the recording session affected the different stimuli equally. This interleaving of stimuli continued until each click intensity had been presented a total of 7480 times (11 clicks/s x 34 s x 20 blocks).

#### 2.5.3 ABR analysis

Differential waveforms were created using Fz-M1 and Fz-M2. In all but three participants these two montages were averaged. For three listeners, one of the montages was confounded by an electrode offset exceeding the criterion, and the other montage was used for the analysis. The two click levels were analyzed separately. The demeaned RMS value of all 7480 sweeps was calculated for each participant using a sweep starting at 17 ms pre-stimulus and ending 17 ms post-stimulus (with the mean calculated over the whole sweep). For each participant, all sweeps which had a broadband RMS power within two standard deviations of their mean were retained for further analysis. These sweeps were averaged in the time domain and the resultant waveform band-pass filtered between 300 and 1500 Hz. This average waveform was then subjected to an automated peak- and trough-picking procedure based on extracting the phase reversals from the first derivative of the time series.

Time windows were constructed around waves I, III and V and the largest peak within the window was selected. The center of the window was determined by the peak in the grand averaged ABR waveform using all 126 participants at each level separately. At 100 dB peSPL, these values correspond to 1.84, 3.85 and 5.74 ms for waves I, III and V respectively. At 80 dB peSPL, they were 2.69, 4.46 and 6.41 ms. The edges of the window were set by using standard deviations of ABR latency reported by Issa and Ross (1995). Standard deviations were 0.17 ms for waves I and III, and 0.21 ms for wave V. The bounds of the windows for our analysis were set as ±3 standard deviations around the peak central values described above. The following trough was constrained to fall within 2 ms of the identified peak. If multiple troughs were present, the one which gave the largest peak-to-trough amplitude was used. If no peak or trough was identified within these constraints, the participant was removed from that specific wave-level analysis. On average, a peak-trough complex which satisfied these criteria was identified 95% of the time. A visual inspection of the automated output confirmed that appropriate peaks from the ABR waveform were being selected.

Differential measures are also informative, to control for individual variability in ABR amplitude and latency due to factors unrelated to synaptopathy such as head size and skull thickness (Picton et al., 1981, Jerger and Hall, 1980). A common method to correct for such confounds is to take a within subject differential measure such as the wave I:V ratio (Schaette and McAlpine, 2011) and inter-peak intervals, such as I-V (Xu et al., 1998). These differential measures taken across different wave peaks are presented in conjunction with differential measures across the two levels. The 100 dB to 80 dB ratio is taken for amplitudes and the 100 dB–80 dB difference is taken for latencies. The two approaches make different assumptions about how synaptopathy affects the human ABR i.e. whether it only attenuates wave I as proposed by Schaette and McAlpine (2011), or whether it targets specific sound intensities.

#### 2.5.4 FFR stimuli

Two contiguous acquisitions were made, with the temporal fine-structure (low-frequency) FFR and temporal envelope FFR (high-frequency) being measured simultaneously.

In each acquisition four tones were presented simultaneously, with a low-frequency tone (240–285 Hz) and a low-frequency tone (240–285 Hz) transposed to 4 kHz (Bernstein and Trahiotis, 2002) presented to each ear. A transposed tone allows the neural firing pattern in a high-frequency region of the cochlea to mimic the firing pattern evoked by a pure tone presented to a low-frequency part of the cochlea. For one acquisition, the left ear received a 255 Hz pure tone and a 240 Hz transposed tone, and the right ear received a 270 Hz pure tone and a 285 Hz transposed tone. For the other acquisition, the left ear received a 285 Hz pure tone and a 255 Hz transposed tone, and the right ear received a 240 Hz pure tone and a 270 Hz transposed tone. Stimuli were 220 ms in duration (including 10 ms ramps) and presented at 80 dB SPL. Each stimulus was presented 4000 times in alternating polarity (2000 repetitions for each polarity) with an inter-stimulus interval randomly selected within the range 85–95 ms.

#### 2.5.5 FFR analysis

The montage used for the analysis was Fz-C7. The use of multiple measurement frequencies allows the calculation of group delay as a measure of response latency. However, the variability in this measure was too high to give a reliable estimate of the latency of the response as there was a large degree of overlap in the complex plane of response to the different frequencies, and so the present analysis focuses solely on the magnitudes of the responses. For each polarity, sweeps were maintained for further analysis if their RMS power was within two standard deviations of the mean. Included sweeps were averaged in the time domain to produce an average for each polarity. These averages were summed to produce a waveform that contains the envelope FFR for the high-frequency region and also subtracted to produce a waveform which emphasizes the fine structure FFR for the low frequency region. A 200-ms window was used for the analysis, which began 10 ms after stimulus onset.

The signal was computed for each component of interest by extracting the magnitude of the fast Fourier transform at the relevant frequency. The noise at each frequency was estimated by using a permutation scheme. Permutation tests are commonly used in electromagnetic recordings to estimate the null distribution of a response (e.g. Maris and Oostenveld, 2007) and although exchangeability of condition labels is a common implementation, for phase-locked signals it has been shown that the phase of each trial can be exchanged in order to build up a null distribution (Prendergast et al., 2011). Before the average was computed for each polarity, half of the sweeps were selected at random and the sign of the response was inverted, which has the effect of making the stimulus polarity arbitrary and any components which remain in the subsequent average can only be spurious in origin. This is repeated 1000 times with different random selections of sweeps to invert. For each permutation the Fourier component of interest is extracted and from this distribution of 1000, the 90^th^ percentile was used to estimate the noise. For both the fine-structure and envelope FFR, the four responses (two from each stimulus/acquisition) were expressed as SNRs and the average of these converted into dB.

## 3 Results

### 3.1 Noise exposures

**Fig. 1:**
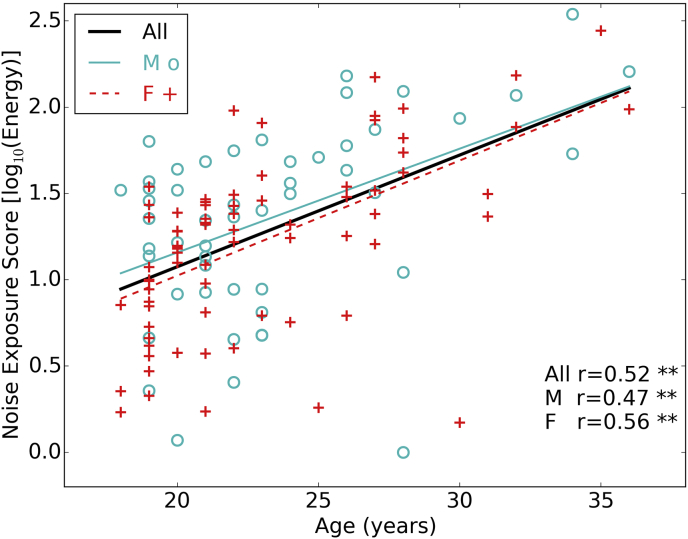
Noise exposure scores as a function of age. 126 individuals are shown, with males (51) and females (75) plotted in different colors and symbols. Regression lines are plotted for the full group and for males and female separately, with the Pearson correlation coefficient shown in the text (* = p < 0.05, ** = p < 0.01). (For interpretation of the references to colour in this figure legend, the reader is referred to the web version of this article.)

Fig. 1 shows estimated lifetime noise exposure scores for all 126 participants as a function of age. Note that the y-axis is a logarithmic scale with respect to energy: the individuals with the highest exposures had about 300 times the lifetime exposure energy compared to those with the lowest exposures. There is no significant difference between noise exposure scores for males (mean = 1.35, s.d. = 0.55) and females (mean = 1.21, s.d. = 0.50): t (124) = 1.48, p = 0.14. The Pearson correlation coefficients presented in Fig. 1 show that noise exposure and age are positively related to each other, which is expected since our noise exposure measure reflects cumulative exposure (p = 3.11e-10 for the full group).

### 3.2 Audiometric data

**Fig. 2:**
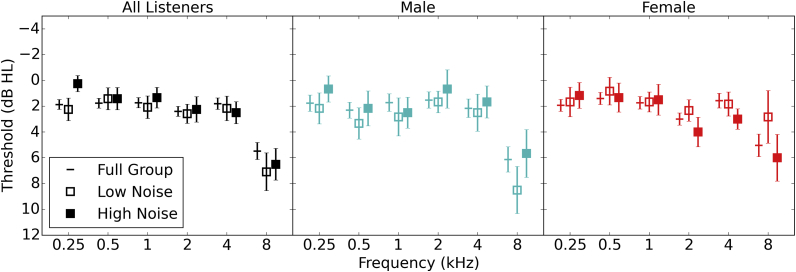
Pure tone audiometric thresholds. Hearing thresholds (averaged across ears and listeners) are shown, with standard errors, for the whole group and also for males and females individually. For all three groups of listeners, the full group is shown as a horizontal line, and the highest and lowest noise exposed individuals are shown as solid and open squares respectively. For all listeners, the low and high noise groups comprise the lowest and highest 30 listeners in terms of noise exposure respectively. For males and females, N = 15 for low and high noise subgroups.

Fig. 2 shows audiometric data in the standard frequency range (averaged across the ears) for all listeners and for males and females separately. In subsequent analyses it is instructive to look at groups of low and high noise exposure, as this provides a useful indication of how well a measure might be able to distinguish listeners with noise induced synaptopathy and those without. Therefore Fig. 2 also shows mean audiometric data for low and high noise exposed groups which were obtained by using the 15 individuals with the lowest and highest noise exposure scores from each sex, and for the group “all” by taking the mean of the 30 lowest and highest noise exposed individuals, regardless of sex. It can be seen that there is very little effect of noise exposure on audiometric threshold for these frequencies.

At 2, 4, and 8 kHz the females with high noise exposure show higher thresholds than the low-noise females as one might expect, whereas for the males this relation is surprisingly inverted, although the differences are not statistically significant. Pearson correlation coefficients were calculated between noise exposure and the average pure tone detection threshold at 2, 4 and 8 kHz. There is no significant relation between audiometric threshold and noise exposure for either males (r = 0.00) or females (r = 0.09), p > 0.05 in both instances.

**Fig. 3:**
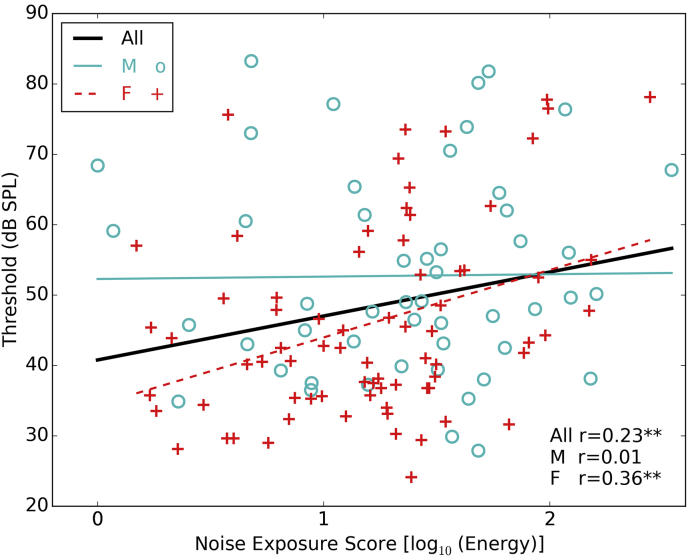
High-frequency (16 kHz) audiometric thresholds. Regression lines are plotted for the full group and for males and female separately, with the Pearson correlation coefficient shown in the text (* = p < 0.05, ** = p < 0.01).

Fig. 3 shows the 16-kHz audiometric data averaged across the two ears. Males exhibit higher 16-kHz thresholds than females, which is consistent with previous reports (Rodriguez Valiente et al., 2014). In our cohort this difference (mean difference of 6.7 dB SPL) is statistically significant: t (124) = 2.64, p = 0.009. There is no relation between 16-kHz thresholds and noise exposure in males, but females show a significant increase in thresholds with increasing noise exposure. Noise exposure, sex and an interaction term were entered into a regression model as predictors of high frequency thresholds, which confirmed a main effect of sex (Beta = −17.89, p < 0.01) and an interaction between sex and noise exposure (Beta = 9.25, p < 0.05).

### 3.3 Otoacoustic emissions

**Fig. 4:**
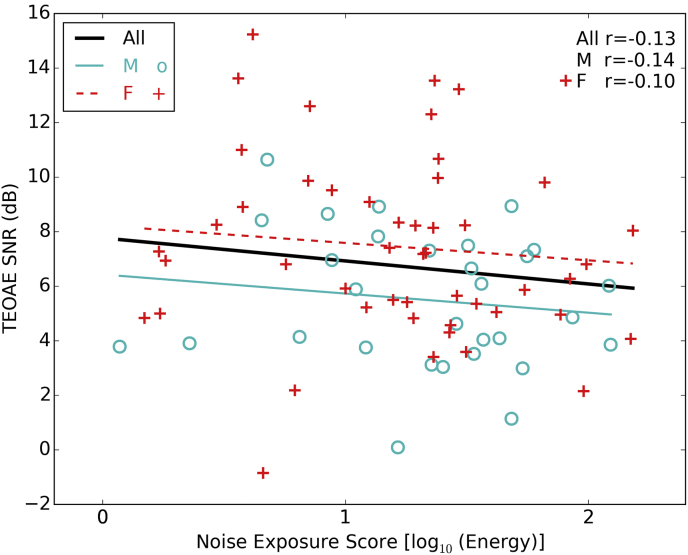
Transient evoked otoacoustic emissions. Males (30) and females (49) are plotted in different colors and Pearson correlation coefficients are shown for both sexes individually and combined. SNRs are the mean across the three test frequencies of 3, 3.5 and 4 kHz and are averaged across ears.

Fig. 4 shows the mean TEOAE SNR averaged between the ears and across test frequencies of 3, 3.5 and 4 kHz. There was no significant relation between noise exposure and the size of the TEOAE (p > 0.05). Although only a subset of participants was able to be included, the data points cover a wide range of noise exposures and suggest that there is little relation between noise exposure and OHC function at the frequencies tested.

### 3.4 ABR

**Fig. 5:**
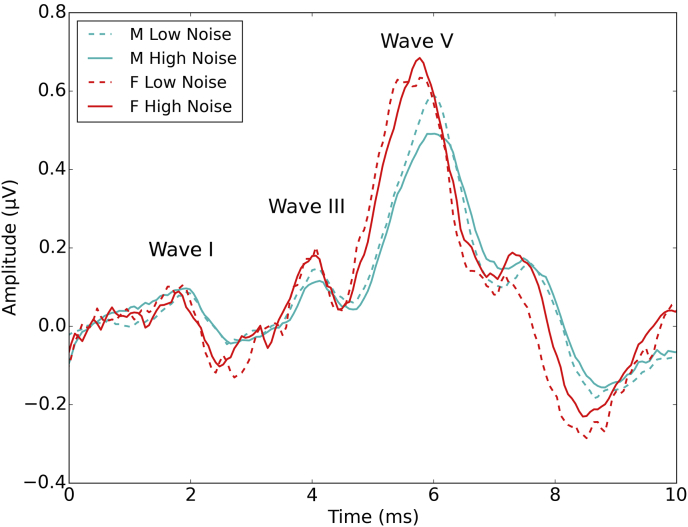
Grand average ABR waveforms. Average waveforms are shown in microvolts for males and females separately and for the 15 lowest and 15 highest noise exposed individuals for each sex. Waves I, III and V can be seen at around 2, 4 and 6 ms respectively. Waveforms are plotted broadband in order to show the full morphology of the response.

Fig. 5 shows grand average ABR waveforms for the low and high noise exposed male and female listeners, for the 100 dB peSPL stimulus. Waves I, III, and V can be readily identified. Females (plotted in red) show larger peak amplitudes and shorter latencies than males. The waveforms for low and high noise exposure groups appear similar.

#### 3.4.1 Amplitude

**Fig. 6:**
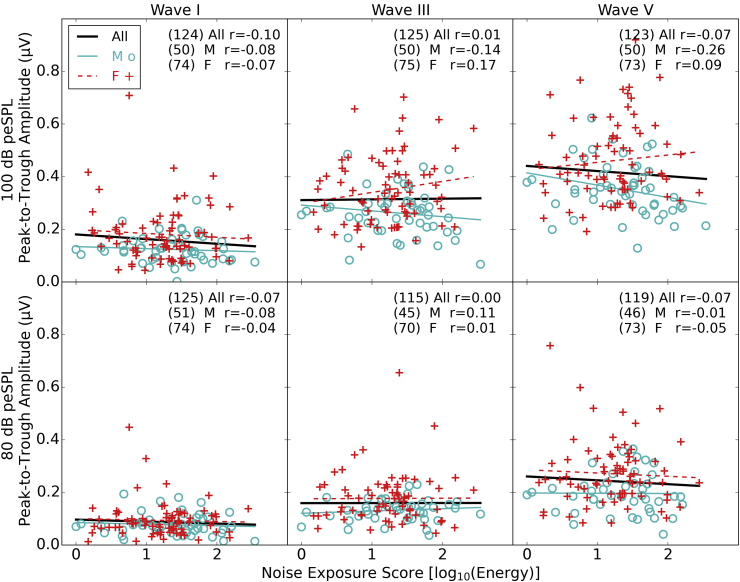
ABR wave amplitudes as a function of noise exposure. The top row shows ABR amplitudes generated by the 100 dB peSPL click and the bottom row those from the 80 dB peSPL click. The columns show the amplitudes of waves I, III and V. Regressions are again plotted for the three groups (all listeners, males and females) with Pearson correlation coefficients shown in the text (* = p < 0.05, ** = p < 0.01). The numbers in brackets are the numbers of participants who produced a measurable peak in each case (see Section 2.5.3).

Fig. 6 shows the peak-to-trough amplitudes of ABR waves I, III and V as a function of noise exposure. The 100 dB peSPL data are plotted on the top row and the 80 dB peSPL data on the bottom row. The ABR amplitudes show the predicted trends as a function of both level and sex, with 100 dB peSPL clicks evoking a larger response for all three waves and females tending to show larger mean amplitudes than males. None of the ABR wave amplitudes vary significantly as a function of noise exposure (Pearson's correlations provided on the figure). For waves III and V, at the higher click intensity, a positive trend is seen in females and a negative trend in males. However, these opposing correlations are not statistically significant.

There is no significant relation between ABR amplitude and the pure tone audiometric threshold averaged across 2, 4, and 8 kHz, for any wave or presentation level. The only relation of note between ABR amplitude and 16 kHz threshold is that for wave III in response to the 80 dB peSPL click in males, wave III amplitude decreasing with increasing threshold (r = −0.38, p = 0.01 uncorrected).

The wave I amplitudes at 80 dB peSPL appear to be very small and this draws into question the extent to which these can be considered representative of the true underlying physiological response. To address this we performed a further analysis to quantify the noise floor. A baseline analysis window was defined in the pre-stimulus period of the 80 dB peSPL ABR, with a window extending 1.02 ms to match the window length used for selecting wave I peaks. The same criteria were used to identify a peak in this arbitrary window, during which no stimulus-evoked peak was expected to be found. Of the 125 listeners with an identified wave I peak-trough complex at 80 dB peSPL, 85 of these (68%) also had a peak-trough complex present in the baseline analysis window that passed the criteria. Of these 85, only 10 listeners showed a response where the baseline noise peak-trough amplitude was greater than the estimate of wave I amplitude. The mean noise exposure scores of these 10 listeners and the standard deviation were comparable to those of the whole group. This analysis suggests that, although some of the wave I amplitudes are weak, in most cases they likely represent some aspect of the underlying neural function. Furthermore in those instances where the response is not greater than the estimated noise level, there is no bias regarding the noise exposure scores of these listeners.

#### 3.4.2 Latency

**Fig. 7:**
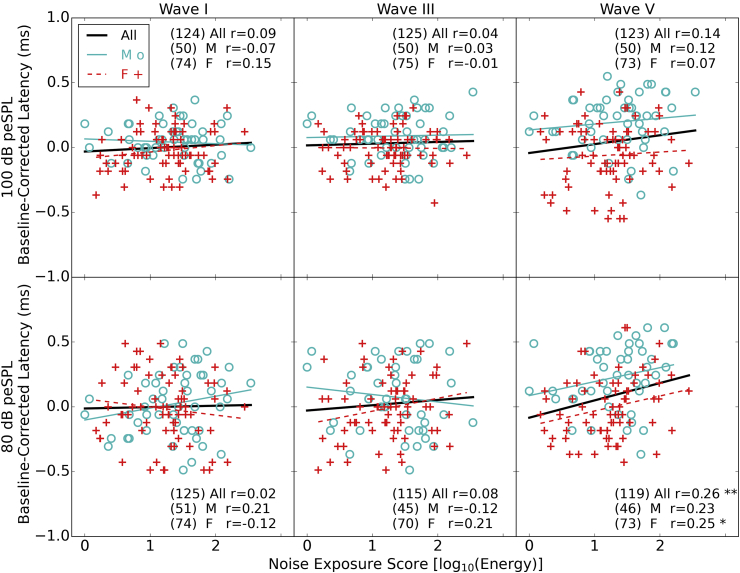
ABR wave latencies as a function of noise exposure. The top row shows ABR latencies generated by the 100 dB peSPL click and the bottom row those from the 80 dB peSPL click. The columns show the latencies of waves I, III and V. All values are baseline corrected so that all three latencies are distributed around zero to allow common axes to be used. The baselines for the 100 dB click were 1.84, 3.85 and 5.74 ms for waves I, III and V respectively. For the 80 dB condition they were 2.69, 4.46 and 6.41 ms. Regressions are again plotted for the three groups (all listeners, males and females) with Pearson correlation coefficients shown in the text (* = p < 0.05, ** = p < 0.01). The numbers in brackets are the numbers of participants who produced a measurable peak in each case (see Section 2.5.3).

Fig. 7 shows the latencies of waves I, III, and V of the ABR to the two click levels used. Values are plotted as “baseline-corrected” latencies, which means that the latency for each individual has been normalized by subtracting a fixed value for each wave (which was the peak latency in the grand averaged waveform across all participants at each level). This allows all the data to be plotted on a single axis for direct comparison. The raw values show previously described trends, with the lower click level evoking waves with longer latencies and females typically showing a shorter mean latency than males.

The upper row shows the latency values for the 100 dB peSPL click, which suggest little relation between noise exposure and ABR peak latency. The regression line for all participants closely matches what is seen in the two sexes independently. For the 80 dB peSPL click, the latencies for wave V are significantly, positively related to noise exposure. Both sexes show the same trend, with the females showing a stronger relation than males. These differences in latency are seen despite the fact that there are no differences in the amplitude of wave V as a function of noise exposure. Furthermore these differences are seen in response to the lower click level rather than the higher click level. These data must be interpreted with care due to the number of contrasts made and the fact that the coefficients have not been corrected for multiple comparisons. In addition, the relation between latency and noise exposure is not significant when age is entered into the model as a predictor: When age is included in the model, neither noise exposure, nor age are significant predictors of latency (Beta = 0.092 and Beta = 0.012 respectively) with an adjusted R^2^ = 0.061.

There is no significant relation between any of the wave latencies and the pure tone audiometric threshold averaged over 2, 4 and 8 kHz. For the 16 kHz thresholds the only relation of interest is with wave V latency for the 80 dB peSPL click in males; latency increasing with increasing threshold (r = 0.35, p = 0.02, uncorrected).

#### 3.4.3 Differential measures

**Fig. 8:**
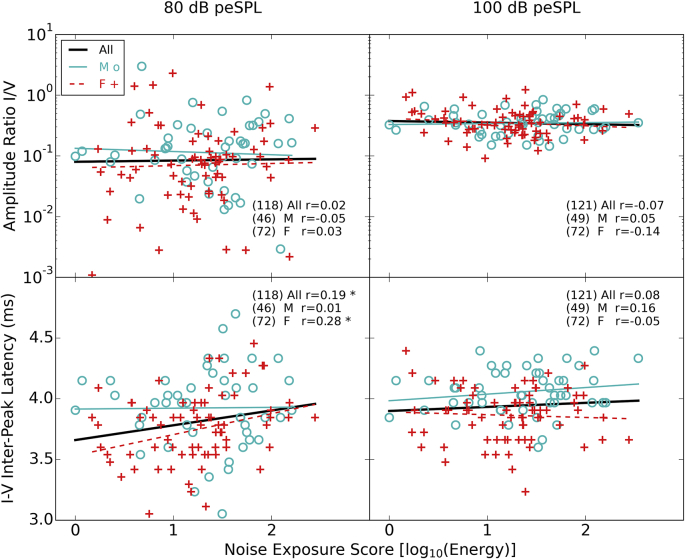
Wave I and V amplitude ratios and latency intervals as a function of noise exposure. The upper row shows amplitude ratios and the bottom row latency intervals whilst the two columns show the values for the 80 and 100 dB peSPL click. Pearson correlation coefficients are shown in the text (* = p < 0.05, ** = p < 0.01). The numbers in brackets are the numbers of participants who produced a measurable peak in each case (see Section 2.5.3).

Fig. 8 shows the difference between waves I and V (expressed as a ratio for amplitude and a difference for latency) for both the 80 and 100 dB peSPL click. There is no significant relation between noise exposure and wave I:V amplitude ratio at either level (p > 0.05). There is a significant relation between noise exposure and wave I-V inter-peak interval at 80 dB peSPL but not at 100 dB peSPL. Given the data presented in Fig. 7 this appears to be driven by a change in the latency of wave V rather than in wave I.

**Fig. 9:**
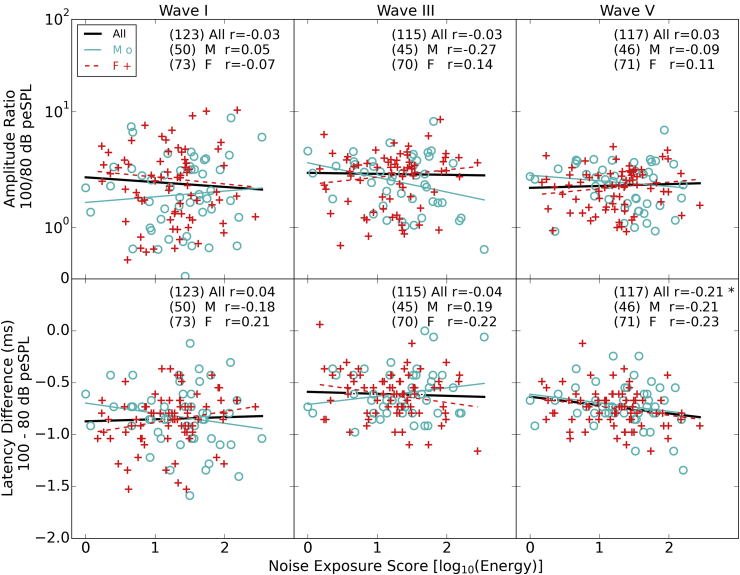
Differential measures with respect to click level as a function of noise exposure. The upper row shows the ratio of amplitudes of the 100 dB peSPL click to the 80 dB peSPL click. The bottom row shows the difference in latency between the peak measured in response to a 100 dB peSPL click and an 80 dB peSPL click. Pearson correlation coefficients are shown in the text (* = p < 0.05, ** = p < 0.01). The numbers in brackets are the numbers of participants who produced a measurable peak in each case (see Section 2.5.3).

In the current study we used two click levels. It was predicted that responses to the 100 dB peSPL click should more affected by noise-induced cochlear synaptopathy than responses to the 80 dB peSPL click. Therefore across-level difference measures might reveal effects of synaptopathy, by reducing between listener variability due to unrelated factors. Fig. 9 shows these differential measures for both amplitude and latency. The amplitude ratios are uncorrelated with noise exposure. The latency data are in agreement with the data seen previously (Fig. 7) when the raw, baseline-corrected values were plotted, with increasing noise exposure resulting in a greater difference in latency across the two click levels for wave V of the response. The driving force behind this differential measure and its relation to noise exposure is a delayed response to the low-level click as noise exposure increases, and not a faster response to the higher-level click.

There is no significant relation between any of the differential measures and the pure tone audiometric threshold averaged over 2, 4, and 8 kHz. For the 16 kHz thresholds, they are predictive of ABR wave III amplitude ratios for the full group (r = 0.18, p = 0.05, uncorrected) and wave V amplitude ratios for the full group (r = 0.24, p = 0.01, uncorrected). In both cases, the ratio increases with increasing threshold. 16 kHz thresholds are also predictive of wave V latency differences at the two levels for both the full group (r = −0.27, p < 0.01, uncorrected) and the males (r = −0.41, p < 0.01, uncorrected). In both cases the latency difference between wave V at the two levels increases with increasing threshold.

#### 3.4.4 Low and high noise subgroups

**Fig. 10:**
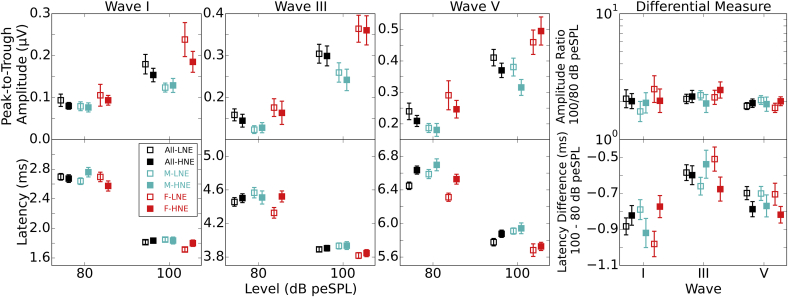
Subgroup analyses of low and high noise exposed individuals. Amplitudes (top row) and latencies (bottom row) are shown for the two click levels. Results for waves I, III and V are shown and the right hand panel plots the differential measures for the three waves. Black symbols represent the full group, with cyan and red showing males and females respectively. The lowest noise exposed individuals are shown as open symbols and the highest noise exposed as closed symbols. Error bars show standard errors.

The linear regression approach assumes that each additional unit of noise exposure produces a constant increase in synaptopathy, which is then reflected in ABR amplitude or latency. However, this approach could be misleading. It may be that a subset of listeners at the upper end of the distribution have exposed themselves to sufficient levels of noise to induce synaptopathy, or it could be the case that in an industrial society only a subset of listeners at the lower end of the continuum have sustained less than a maximum degree of synaptopathy. To address this, Fig. 10 shows the differential latency and amplitude measures for just the upper and lower parts of the noise exposure distribution using the same selection criteria as for Fig. 3. In general the plots are consistent with the results of the previous correlation analyses, showing little effect of noise exposure.

### 3.5 FFR

**Fig. 11:**
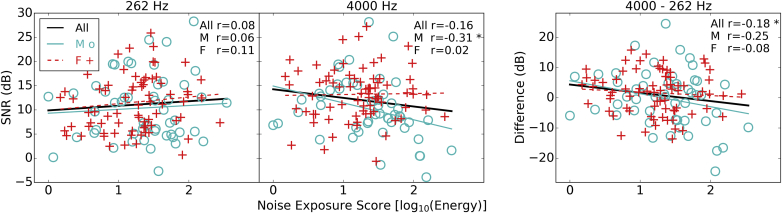
FFR SNRs as a function of noise exposure. The left panel shows SNRs in response to the low frequency pure tones (average SNR across the four frequencies used: 240, 255, 270 and 285 Hz). The middle panel shows SNRs to the high frequency transposed tone (average SNR across the modulators of a 4 kHz carrier: 240, 235, 270 and 285 Hz). The right-hand panel shows the difference between the two. Pearson correlation coefficients are shown in the text (* = p < 0.05, ** = p < 0.01).

Fig. 11 shows the SNR of the FFR as a function of noise exposure. Phase-locking to a low-frequency pure tone (240–285 Hz) and to a 4-kHz carrier amplitude modulated at 240–285 Hz were measured based on the assumption that noise-induced synaptopathy would affect temporal coding in the high frequency region but not the low frequency region. A differential measure is shown in the right-sided panel of Fig. 11, computed in an attempt to reduce the variability from sources other than synaptopathy. The plotted regression lines and reported correlation coefficients indicate that the FFR for the low-frequency region did not vary greatly as a function of noise exposure, with comparable responses seen across males and females (p > 0.05). The FFR for the high-frequency region, evoked by envelope fluctuations, shows a significant decrease in SNR as a function of noise exposure in males, whereas females show little relation between FFR signal-to-noise ratio and noise exposure. However, the interaction between sex and noise exposure is not significant (p = 0.056). Furthermore, when age is entered into the model, noise exposure no longer singificantly predicts the strength of the envelope FFR in male listeners (Beta = −2.98) with an adjusted R^2^ value of 0.067. The differential measure taken between low and high frequency FFRs shows a negative correlation across the whole group, though this effect is weak and does not survive correction for multiple comparisons.

## 4 Discussion

In our large cohort of audiometrically normal young adults, there is no evidence that the amplitudes of sub-cortical electrophysiological measures of auditory coding are attenuated substantially due to noise exposure. Hence, the data do not support the hypothesis that cochlear synaptopathy varies as a function of lifetime noise exposure in young adults. There are, broadly speaking, three possible explanations for our results:

1. Noise-induced cochlear synaptopathy is not prevalent in young audiometrically normal adults;
2. Noise-induced cochlear synaptopathy is prevalent in young adults with comparatively low exposures and there is no additional consequence of higher levels of exposure; or
3. Our measures are insensitive to cochlear synaptopathy in humans

There are a number of factors that affect the likelihood that each of these three explanations is correct. These are discussed below.

### 4.1 The role of high frequency thresholds

The aims and methods of the present study are similar to those described by Stamper and Johnson (2015a), except that we had a larger sample and used a lifetime measure of exposure rather than a measure over the previous year. We did not replicate the decrease in ABR wave I amplitude as a function of noise exposure reported in that study. There was a potential confound of sex in the original presentation of their data and this was followed up with a letter to clarify how sex interacts with the reported trend (Stamper and Johnson, 2015b), with an effect of exposure demonstrated for females but not for males (and only reported for the very highest click level of about 120 dB peSPL). However, we did not find an effect of noise exposure on ABR amplitudes for either sex. The ABR amplitudes in the present study are smaller than those reported by Stamper and Johnson for a comparable click level (partly due to the narrowband filtering used here to facilitate the automatic peak-picking procedure). However, the amplitudes in the current study are consistent with those reported by Schaette and McAlpine (2011).

One explanation for the discrepancy between the present study and Stamper and Johnson, 2015a, Stamper and Johnson, 2015b is the potential confound of high-frequency hearing loss. The ABR is predominantly generated by AN fibers with high characteristic frequencies (Abdala and Folsom, 1995). The frequency response of the ER3A transducer used in both our study and that of Stamper and Johnson rolls off significantly above about 4 kHz. In the Stamper and Johnson study, audiograms were matched across noise exposures up to 8 kHz. However, presenting very high click levels of about 120 dB peSPL (as used by Stamper and Johnson, 2015a, Stamper and Johnson, 2015b) will cause significant spread of excitation to the basal cochlear region. Furthermore, it is unclear from the report of the follow-up analysis (Stamper and Johnson, 2015b) whether audiograms up to 8 kHz were matched across noise exposures for the sexes independently. Therefore the extent to which loss in sensitivity at very high frequencies could account for the effects of noise exposure on ABR amplitudes is unclear.

In our study, which used a 100 dB peSPL click, the spread of excitation will be less extensive and therefore these high frequency regions may contribute less to the response. Due to the basalward half-octave shift of the traveling wave at high levels (McFadden, 1986), the stimulus at the output of the ER3A insert transducer was likely providing maximum excitation for characteristic frequencies between about 2.25 and 6 kHz. Our assumption was that the spectral region most susceptible to synaptopathy is the same as the region most susceptible to noise-induced audiometric hearing loss in humans, i.e., the 3 to 6 kHz region (Toynbee, 1860, McBride and Williams, 2001). If synaptopathy in humans manifests at a different spectral region then it may be that alternative, perhaps wider-band, stimuli would provide more sensitivity for detecting its presence. It is also worthy of note that the environmental noise humans are typically exposed to has a wider bandwidth than the noise used in rodent studies of synaptopathy, and thus this may reduce the likelihood of causing synaptopathy in any given frequency region.

In our 16-kHz audiometric data females showed a greater effect of noise exposure than males, with the high noise females showing poorer high frequency sensitivity than low-noise females. If very high frequency contributions to the ABR account for the differences in wave I between high and low noise exposure groups, then our data suggest that this would occur in females but not males, which is the pattern reported in the follow-up analysis of Stamper and Johnson (2015b).

It is important for future research studies to control for the effect of high frequency hearing sensitivity, but it is also worth considering the potential clinical utility of high frequency audiometry (above 8 kHz). High frequency thresholds may provide an early marker of noise-induced damage to the auditory system. Furthermore, in our cohort the relation between lifetime noise exposure and high-frequency sensitivity was significantly greater for females than for males, which suggests different vulnerability of the basal cochlear region in the two sexes.

### 4.2 Does noise-induced cochlear synaptopathy occur in young audiometrically normal humans?

Despite the large sample size, the data collected in the present study provide no evidence for the existence of noise-induced cochlear synaptopathy in listeners with normal audiometric thresholds. However it is possible that noise exposure does cause synaptic changes in these listeners, but that these effects are subtle and within the range of expected inter-subject variability. It may also be the case that in an urban environment, a large majority of individuals have already sustained a comprehensive noise-induced loss of low-SR fibers and therefore our measures are reflecting a minimal residual response across all exposures. An argument against this latter hypothesis is that temporal bone studies suggest a progressive loss of spiral ganglion cells across the lifespan, rather than an abrupt loss at a young age followed by no further decline (e.g. Makary et al., 2011). Furthermore it is generally accepted that the ABR reaches maturity by the age of around 2 years in humans, at which point the amplitudes and latencies are comparable to those seen in adulthood (Hecox and Galambos, 1974). If noise-induced synaptopathy was affecting ABRs on a large scale prior to the ages tested in the current study, there would be a clear reduction in response sometime after maturation, and this is not the case.

Although the rodent model of cochlear synaptopathy is compelling, it may be that humans are physiologically less vulnerable to noise-induced synaptopathy than rodents. It could also be the case that the noise exposures used in the rodent work are not representative of an equivalent human exposure. Kujawa and Liberman (2009) showed temporary threshold shifts, in response to 2 h of 100 dB SPL noise, of 40 dB one day post-exposure and 20–25 dB three days post-exposure in the ABR measured at 3 and 5 kHz. For comparison in humans, Howgate and Plack (2011) report a 10.8 dB temporary threshold shift at 4 kHz immediately after attending a music venue with a mean equivalent exposure level of 99 dBA. It may be that cochlear synaptopathy in humans only occurs for exposure levels close to or greater than those that produce a permanent threshold shift. Noise levels can be titrated in the rodent model, but the likelihood of finding a human listener who has been exposed to noise levels that produce synaptopathy without leading to permanent threshold shift may be very small. In other words, in humans noise-induced synaptopathy may not exist without a permanent threshold shift. By focusing on listeners with audiometric thresholds within the normal range, we may have been selecting listeners who were not synaptopathic. Another unknown issue in humans is the extent to which vulnerability varies across listeners. In the rodent models of synaptopathy, there is little or no genetic variation, nor substantial differences in life experience prior to the experimental procedures. In human listeners it is unknown whether the susceptibility to synaptic loss is equivalent across the sexes, across the lifetime, or across different listeners with the same age and sex. The notion of “tough” and “tender” ears has long been considered in the context of noise-induced hearing loss (Cody and Robertson, 1983) and a similar concept may be applicable for noise induced cochlear synaptopathy.

Even if the noise levels humans are typically exposed to are sufficient to cause synaptopathy, there may be complex and co-dependent changes as a function of age. It has also been shown recently that noise exposure at a young age in rodents accelerates age-related synaptopathy (Fernandez et al., 2015), although the inter-play between noise exposures and age remains unclear even in rodents. Therefore, it may be that humans are robust to synaptopathy until age-related changes take effect on the auditory system, or that noise exposure early in life changes the likelihood of rapid auditory decline later in life. In addition, it is possible that in humans the initial loss is to the low-SR fibers implicated in the animal work, but that the loss progresses to lower-threshold fibers with increased exposure and/or age. If the low-SR fibers have a small contribution to wave I, as suggested by Bourien et al. (2014), then the effects of exposure on wave I may be more evident in older listeners. By focusing on young and healthy listeners it may be that these subtle effects cannot be reliably identified. However, if this is the case then it may prove difficult to resolve the contribution of synaptopathy and the loss of sensitivity due to age-related hair-cell dysfunction when both are present. Such an account, where age is a crucial modulator of the effects of noise exposure, could account for the largely null findings in the current study despite Schaette and McAlpine (2011) and Gu et al. (2012) reporting attenuated wave I responses in humans, as these previous studies used listeners that were on average 10 years older than the cohort in the current study.

Much of the early work on noise-induced synaptopathy was conducted in mice, for whom the loss of cochlear synapses appears to be irreversible. However, comparable noise-exposure studies in guinea pigs have suggested that, after an initial reduction in the number of presynaptic ribbons, the synapse count may largely recover (Liu et al., 2012, Shi et al., 2013). It appears as though these synapses are reformed to some degree, but although they are present, their coding properties are functionally abnormal, both in their amplitude and latency profiles (Song et al., 2016). These studies suggest clear differences in the manifestation of cochlear synaptopathy in the guinea pig compared to the mouse and they also report ribbon damage to high-SR units as well as the more widely demonstrated loss of low-SR fibers. Therefore, given the marked cross-species differences between noise-induced synaptopathy in the mouse and the guinea pig, we must be cautious in our expectations of how cochlear synaptopathy may present itself in the human listener.

### 4.3 Are the measures sufficiently sensitive to detect synaptopathy?

Measurement variability in the human listener is a serious problem when investigating subtle differences in electrophysiological measures. The rodent results, which have motivated the search for synaptopathy in humans, are based on direct observations of synaptopathy using histological techniques. In human listeners, the most direct non-invasive measure of synpatopathy, wave I of the ABR recorded via scalp-mounted electrodes, is highly variable across individuals (Beattie, 1988, Lauter and Loomis, 1988).

Bourien et al. (2014) used ouabain to selectively destroy AN fibers in the gerbil in order to investigate the contribution of low-, medium-, and high-SR fibers to the compound action potential (CAP), which is a measure of the AN response comparable to ABR wave I. Low-SR fibers were the most susceptible to damage via ouabain and it was found that even when this fiber group was greatly depleted, the CAP did not reduce substantially. These results suggest that low-SR fibers contribute little to the CAP (probably due to their delayed, and broadened, first spike distribution), and by implication to ABR wave I. This account is somewhat contradictory to the findings of Schmiedt et al., 1996, Furman et al., 2013 in which loss of predominantly low-SR fibers was shown to attenuate the AN response (other fiber groups were also possibly affected). Bourien et al. (2014) suggest that this contradiction may be related to whether fibers are classified into three groups or just two, with medium-SR fibers grouped in with the low-SR fibers. Medium-SR fibers do seem to be affected by noise-induced synaptopathy (Furman et al., 2013) and hence ABR wave I would still be expected to be reduced by synaptopathy. However, if fibers with the lowest SRs do not contribute to wave I, the sensitivity of this measure could be limited. Bourien et al. (2014) also highlight the fact that the distribution of fiber types as a function of frequency varies across species and therefore our assumptions of the fiber groups and their relative distributions in humans may be inaccurate.

Recently, Mehraei et al. (2016) demonstrated that the change in the latency of wave V with increasing masking noise level mimics the drop in amplitude of wave I. Low-SR fibers have a longer response latency but are more resistant to noise masking. Hence the effect of low-SR fiber loss is hypothesized to be a reduction in the latency increase with increasing background noise level. Therefore although the variability of wave I makes its suitability as a diagnostic tool uncertain, it may be that the reduced response of auditory nerve fibers as a result of cochlear synaptopathy can be reliably inferred by measuring the response further along the ascending auditory pathway. It remains unclear whether the wave V metric described by Mehraei et al. (2016) is related to lifetime noise exposure.

The FFR has been suggested as a reliable alternative to the ABR with which to evaluate the temporal coding of the auditory periphery (Shaheen et al., 2015, Bharadwaj et al., 2015). The FFR paradigm utilized in the current study assessed the ability of the auditory system to phase lock to low-frequency pure tones and to the modulated envelope of a high-frequency pure tone carrier. A pilot to the project suggested that contrasting FFRs from low- and high-frequency regions is able to differentiate between individuals with high and low levels of noise exposure (Barker et al., 2014). In the current study this measure showed a weak relation with noise exposure for the envelope following response in the high frequency region, but only in male listeners. The differential measure showed a weak relation in the hypothesized direction for all listeners combined, but this result must be approached with caution as it appears as though it may be driven more by the male listeners than the female listeners, even though this interaction does not reach significance in the current cohort.

Bharadwaj et al. (2015) described an FFR approach which uses different depths of modulation presented in a notched masking noise. Again, the aim of this approach was to accentuate the contribution of low-SR fibers to the response by including high levels and low modulation depths so that the dynamic range of the level fluctuations was above the saturation level of the high-SR fibers. An FFR was measured to modulation depths ranging from 0 to −20 dB and the slope of this function (SNR vs modulation depth) in the range −8 to 0 dB was shown to be predictive of performance on a number of auditory perception tasks. Rudimentary information was collected on listeners' noise exposure history and this analysis suggests that the slope of the function which describes how the FFR changes as a function of modulation depth could be sensitive to underlying noise-induced cochlear synaptopathy.

A further potential cause of low sensitivity to the effects of noise exposure comes from the noise estimation process itself. The approach used in the current study relies on a subjective recall of both current and historical noise exposures to high-intensity sound. Such a measure will undoubtedly be affected by recall errors and bias. Such errors are potentially exacerbated in older listeners as they are required to recall further into the past, and therefore may grossly under- or over-estimate the frequency with which certain activities were performed. In the cohort studied in this work, many of the younger people were able to confidently estimate the frequency of their attendance at high-noise events as they are still in the habit of going to these events and could often think in terms of distinct periods of time such as years spent at school, college, or university. The older listeners in this cohort typically worked in high-noise environments and many of these were able to clearly describe their working patterns as they moved around different jobs and venues and, as it was occupational rather than recreational noise, they were much more aware of the frequency and duration of time spent in high-noise environments. However, despite these mitigating factors, a subjective recall of noise exposure remains an undesirable measure to use as the main predictive factor of an underlying pathology. Unfortunately, for human studies there is no method that is able to reliably and accurately capture the information that is required retrospectively. While this potential lack of accuracy should not be overlooked, it is important to emphasize that the differences in estimated exposure were so great between the lowest and highest exposed in the current cohort that it is unlikely that meaningful effects were washed out by variability in the estimates.

### 4.4 Effect of noise exposure on ABR latency

One positive finding is the increase in ABR wave V latency as a function of noise exposure for the 80 dB peSPL click. An increase in latency could reflect a reduction in the contribution of short-latency basal generators to the ABR. However, the fact that this relation occurs only for wave V and only for the lower-level click condition and not for the higher-level click does not fit easily with the low-SR model of cochlear synaptopathy. Furthermore, given that the latency of low-SR fibers is greater than that of high-SR fibers (Rhode and Smith, 1985), it is also not clear that loss of low-SR fibers would produce an increase in latency, rather than a reduction.

It should be noted that the effect of latency did not survive control for age. This is not surprising, given that age is strongly related to lifetime noise exposure in our cohort, such that it is difficult to disentangle the effects of the two. Regardless of how often a young individual goes to high noise events, they will always struggle to match the noise exposures of individuals with 10 years more life experience. However, it is possible that age *per se*, rather than noise exposure, is causally related to latency. Given that the participants are audiometrically homogeneous, it is not clear what aspect of ageing underlies this increase in latency. Previous studies have shown an effect of age on ABR latency and amplitude (Konrad-Martin et al., 2012), although it is unclear to what extent cumulative noise exposure could be a contributing factor.

## 5 Conclusions

1. In a large group of young, audiometrically normal, human listeners, there was no relation observed between noise exposure and mean ABR amplitude. Contrary to rodent models, the ABR wave I results provide no evidence for noise-induced cochlear synaptopathy in the young human cohort studied. It remains possible that the effects of exposure are more evident in older individuals, or are more easily observed at higher characteristic frequencies than the 3–6 kHz region on which this study primarily focussed.
2. The amplitude of the envelope FFR for a high frequency carrier decreased with increasing noise exposure, but the relation was weak and was only observed for male and not for female listeners.
3. 16-kHz audiometric thresholds increased with noise exposure for females but not for males, indicating a possible sex difference in vulnerability to the effects of noise.
